# A Case Report on the Atypical Symptoms of the Synovitis, Acne, Pustulosis, Hyperostosis, and Osteitis (SAPHO) Syndrome: Could COVID-19 Be a Cause?

**DOI:** 10.7759/cureus.41498

**Published:** 2023-07-07

**Authors:** Saira E Anwer Khan, Umaima M Khattak, Maira D Nousherwani

**Affiliations:** 1 Rheumatology, Shalamar Hospital/Shalamar Institute of Health Sciences, Lahore, PAK; 2 Internal Medicine, Shalamar Hospital/Shalamar Institute of Health Sciences, Lahore, PAK

**Keywords:** seronegative, covid complications, etanercept, autoimmune diseases, metatarsals, sterile osteomyelitis

## Abstract

Synovitis, acne, pustulosis, hyperostosis, and osteitis (SAPHO) syndrome, a rare disorder with a spectrum of manifestations and overlapping osseous and cutaneous symptoms, shares pathogenesis with various autoimmune diseases. SARS-CoV-2 has been previously linked to various autoimmune diseases like Guillain-Barré syndrome (GBS), a multi-inflammatory syndrome in children (MIS-C), or rheumatoid arthritis, but there is no existing work showing a link between SAPHO syndrome and COVID-19 yet.

Here, we present a case of a middle-aged Asian male who presented with minimum swelling of his right second toe, 21 days post-COVID. After a series of investigations, namely, MRI scans, 99mTc-methylene diphosphonate three-phase bone scan, and bone biopsy, followed by a positive culture and sensitivity test of the same toe, a trial of vancomycin was given to the patient to treat bacterial osteomyelitis. This resulted in no improvement, pointing toward a misdiagnosis. A conclusion of sterile osteomyelitis of his right second and third metatarsal heads and phalanges due to SAPHO syndrome, as a possible complication of SARS-CoV-2 infection, was made. There are a number of classification systems for diagnosing this syndrome, one of which was modified by Kahn and was used in our case.

Atypical presentations of rare disorders like SAPHO syndrome and their relation to SARS-CoV-2 infection are still to be fully discovered and investigated. Their prevention, timely diagnosis, and management may help in alleviating the discomfort and fear associated with the unknown for the patients.

## Introduction

A deadly virus that initially caused severe pneumonia became a pandemic worldwide in December of 2019 and was officially termed coronavirus disease by WHO the next year [1]. There is a lot of published work explaining the auto-inflammatory nature of COVID-19, aberrant cytokine production, and an unfavorable immunological response [1]. Many autoimmune diseases share COVID-19's inflammatory immune response nature [1]. Not only this but other viruses are also known to disrupt the body's immunity through various methods like molecular mimicry or epitope spreading, herpes virus being linked to systemic lupus erythematosus (SLE), rheumatoid disease, and adult-onset Still disease [1]. Similarly, patients with COVID-19 have been documented to develop antiphospholipid syndrome, autoimmune cytopenia, Guillain-Barré syndrome (GBS), and Kawasaki disease [1]. Among these autoimmune diseases, SAPHO has not been documented as a post-COVID complication, until today.

A rare auto-inflammatory illness known as synovitis, acne, pustulosis, hyperostosis, and osteitis (SAPHO) syndrome may manifest as cutaneous, osseous, or articular abnormalities [2]. The acronym SAPHO was first coined by Chamot et al. in 1987 to acknowledge the combination of these five pathologies that are often present together [3]. Given the overlapping clinical characteristics SAPHO shares with illnesses like chronic recurrent multifocal osteitis (CRMO) and other spondyloarthritides, it is believed that SAPHO belongs to a spectrum of auto-inflammatory disorders [2]. The syndrome has a long-term relapsing and remitting history and can manifest at any age but frequently does so between infancy and middle life [3].

The two primary pathogenic explanations for the syndrome are *propionibacterium* acnes infection and genetic predisposition. Proinflammatory cytokines, such as tumor necrosis factor, are also thought to play a role in SAPHO syndrome in addition to these [4].

Bone hyperostosis induced by sterile osteitis and osteomyelitis, with or without skin involvement, is the primary clinical feature of SAPHO syndrome [5], the presence of which is sufficient for a radiologist to at least evaluate the diagnosis of SAPHO syndrome. It can be severely painful. Numerous joints, including the sternoclavicular, costoclavicular, and anterior chest wall joints, can be impacted by synovitis in this disease and display radiological abnormalities similar to those seen in seronegative spondyloarthropathies. Another extrathoracic location that may be affected is the sacroiliac joint [6].

Palmoplantar pustulosis (PPP), severe forms of acne (acne fulminans or conglobata, hidradenitis suppurativa), and various kinds of psoriasis, particularly pustular psoriasis, are instances of cutaneous symptoms [3].

SAPHO syndrome is generally an innocuous condition, and an effective approach to its treatment is symptomatic relief. SAPHO patients' well-being is likely to improve with early detection and treatment. There are no standardized management protocols available because it is a rare condition. Biological agents, disease-modifying antirheumatic drugs (DMARDs), bisphosphonates, and non-steroidal anti-inflammatory medications are used in the course of symptomatic treatment. These options offer varying degrees of relief [7].

Here, we present a rare case of SAPHO syndrome as a possible post-COVID complication and a delayed diagnosis presenting with non-bacterial osteomyelitis of metatarsals that is characteristic of this syndrome.

## Case presentation

**Figure 1:**
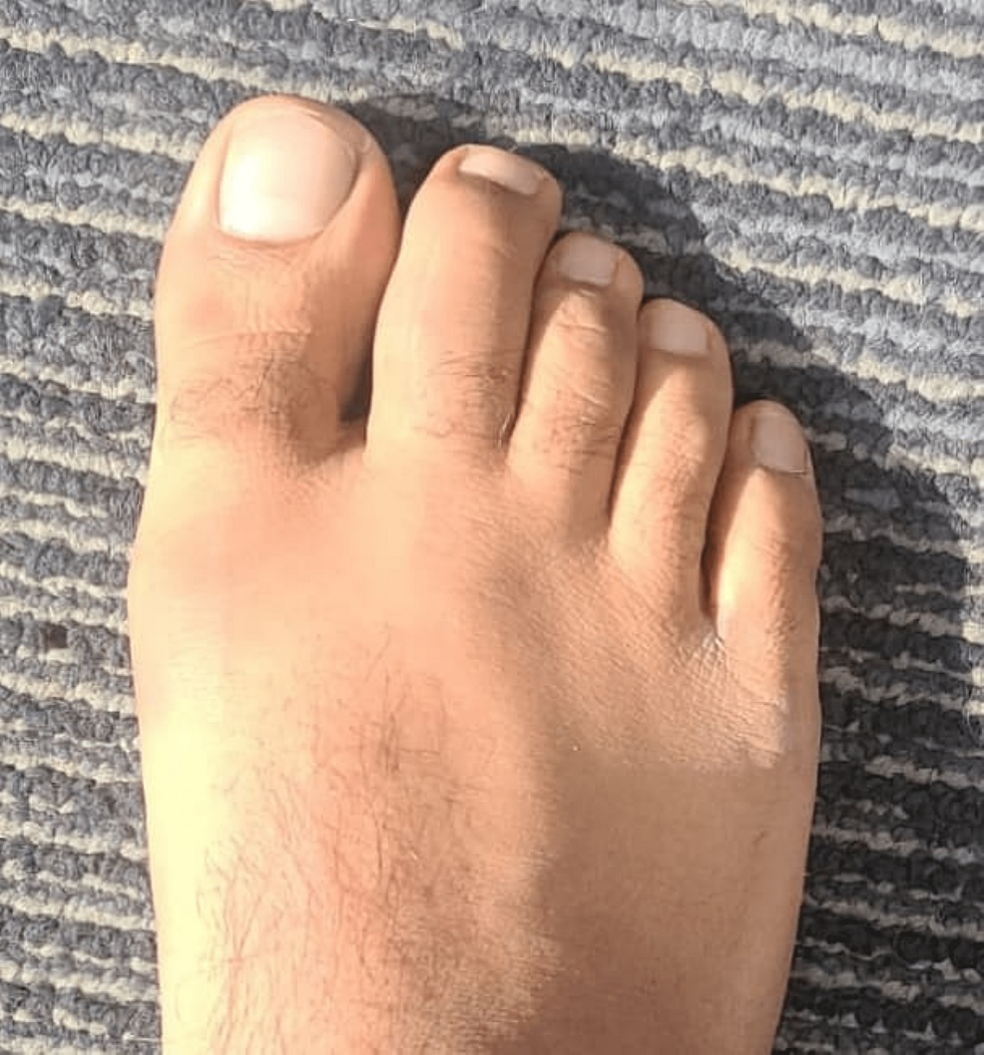
Swelling of the second and third MTP and proximal phalanges of the right foot

A 40-year-old, Asian male recovered from a mild case of SARS-CoV-2 (as categorized according to the National Institutes of Health [NIH] clinical spectrum for SARS-CoV-2) in mid-June 2020. In the following month, he developed pain in his right foot on weight bearing. This happened 21 days after he had been quarantined. The pain was localized to the second and third metatarsophalangeal heads (MTP) and proximal phalanges of the right foot, associated with minimal swelling of the second toe (Figure 1).

He was then seen by a rheumatologist, and his serology and inflammatory markers were investigated including C-reactive protein (CRP)/erythrocyte sedimentation rate (ESR), rheumatoid factor, anti-cyclic citrullinated peptide (anti-CCP) antibody, and HLA-B27 (Table 1). All the results were normal. There was only a slight increase in uric acid levels, i.e., 6.7 mg/dL at that visit. With these findings and lab work, a diagnosis of crystal arthropathy was suspected, and he was prescribed NSAIDs (celecoxib 200 mg every 12 hours for two weeks) followed by a shot of 60 mg methylprednisolone intramuscularly with a 10 mg second shot given via intra-articular route at the second MTP, on the same day. The patient's symptoms did not improve.

**Table 1:** Trends in laboratory investigations *Tests like rheumatoid arthritis factor and anti-CCP were done twice with negative results in both instances. **Normal values have been included. On 16/1/2021, no growth was seen on blood cultures.

Labs (**)/time elapsed since disease onset (June 2020)	3 months	4 months	7.5 months	8 months	8.5 months
ESR mm/first hour (less than age/2 mm/hour)		16	14		
Hb g/dl (13-17 g/dL for men)	14.3	15.9	14.8	15.2	15.2
RBC x10^12/l (4.0 to 5.9 x 10*12/L)	4.66	5.17	5.11	5.23	5.08
WBC x10^9/l (4-10 x 10^9/L)	8.8	7.6	7.23	7.8	8.5
Platelets x10^9/l (150-400 x 10^9/L)	278	320	219	343	284
CRP mg/L (<3 mg/L)		2.36	3.3	4.42	
Uric acid mmol/L (0.18-0.48 mmol/L)		4.5			5.2
Bilirubin mg/dL (<0.3 mg/dL)			0.25	0.3	
ALT U/L (5-30 U/L)			54	32	
AST U/L (5-30 U/L)			56	30	
eGFR mL/min/1.73m2 (60 mL/min/1.73 m2 or more)			102	>60	109
Creatinine mg/dl (0.8-1.3 mg/dL)			0.83	0.9	0.83

**Figure 2:**
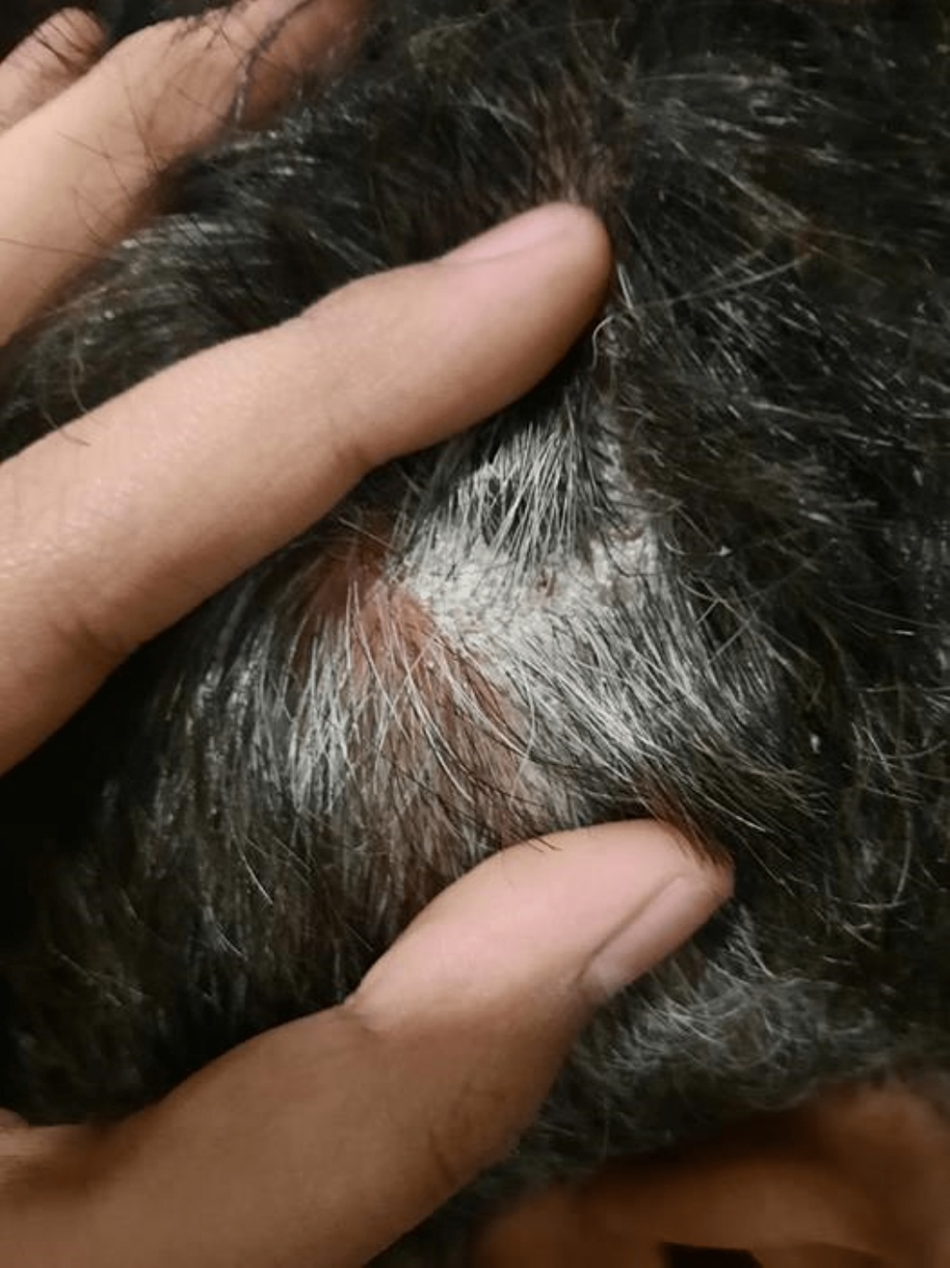
Psoriatic plaques on the scalp (side of scalp)

The rheumatologist also examined the patient's skin and incidentally found psoriatic plaques on the scalp (Figure 2) and pustular acne on his forehead in the absence of other skin conditions or nail changes. The diagnosis was confirmed by a dermatologist later. These skin manifestations were not related to his toe swelling, did not bother him, and were present long before he had COVID, although the acne worsened right after COVID. Some discomfort was felt in the lateral aspect of the left thigh along with an episodic lockjaw, radiologically investigated with normal results. The patient had felt undue stress for the past two years which later coincided with the painful swelling of his MTP.

**Figure 3:**
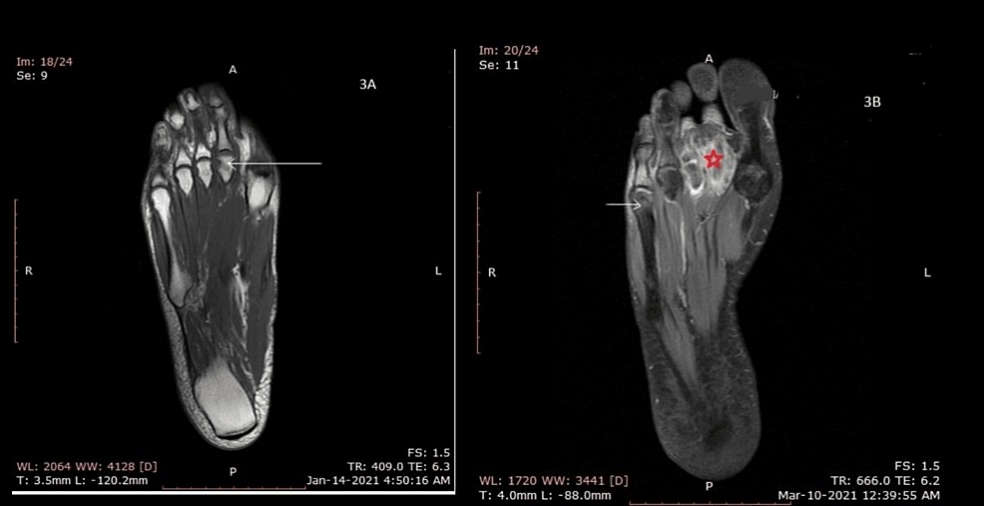
A: An abnormal signal (low T1, high T2) on the head of the second/third MTP (white arrow), proximal/intermediate phalangeal bone. B: Increase in abnormal MR signals in the head of second and third metatarsal bones, proximal and intermediate phalanges (red star). Interval extension around the fourth and fifth head of the metatarsal bones (white arrow)

Three months later, he made homeopathic visits without any improvement in his pain. Later, he took an orthopedic surgeon’s consultation who ordered the first MRI of his right foot. An abnormal signal (low T1, high T2) on the head of the second/third MTP, the proximal/intermediate phalangeal bone, was evident on this MRI pointing toward a possible diagnosis of osteomyelitis (Figure 3A).

Next, a 99mTc-methylene diphosphonate three-phase bone scan was performed which showed increased blood flow and pooling of the radiotracer in the distal part of the right foot proximal phalanges. Delayed images of the scan also showed increased tracer uptake in the distal parts of the second and third metatarsal bones and proximal phalanges.

To further help with the diagnosis, a bone biopsy of the right foot was ordered, and it showed minimal signs of chronic inflammation and fibro-osseous/fatty tissue with foci of myxoid degeneration but no signs of malignancy or granuloma.

In the same month, tissue was sent for culture and sensitivity (C/S) showing moderate growth of MRSA after 24 hours of incubation at 37°C. AFB and ZN stains were negative. These culture reports led to a breakthrough diagnosis of bacterial osteomyelitis, and the patient was started on vancomycin 1 g intravenously twice a day for six weeks.

A repeat MRI was performed after two months, and a comparison was made with a previous MRI which showed an interval increase in abnormal MR signals (low on T1, high on T2, and STIR images with post-contrast enhancement) seen in the head of the second and third metatarsal bones, proximal and intermediate phalanges, extending to the distal phalanx of the second toe, surrounding the soft tissues of the second and third metatarsal and phalangeal bones with an interval extension around the fourth and fifth head of the metatarsal bones, in the fat pad region inferior to the distal first metatarsal bone (Figure 3B).

Despite following the treatment protocol for bacterial osteomyelitis, a repeat MRI (in March) showed worsening of the condition indicating possible contamination, during the previously performed biopsy, of the tissue that was sent for C/S. A misdiagnosed infectious osteomyelitis and overall findings were highly concerning for us to consider another differential diagnosis, sequelae of inflammatory etiology, i.e., sterile osteomyelitis with evidence of underlying bones being involved.

**Figure 4:**
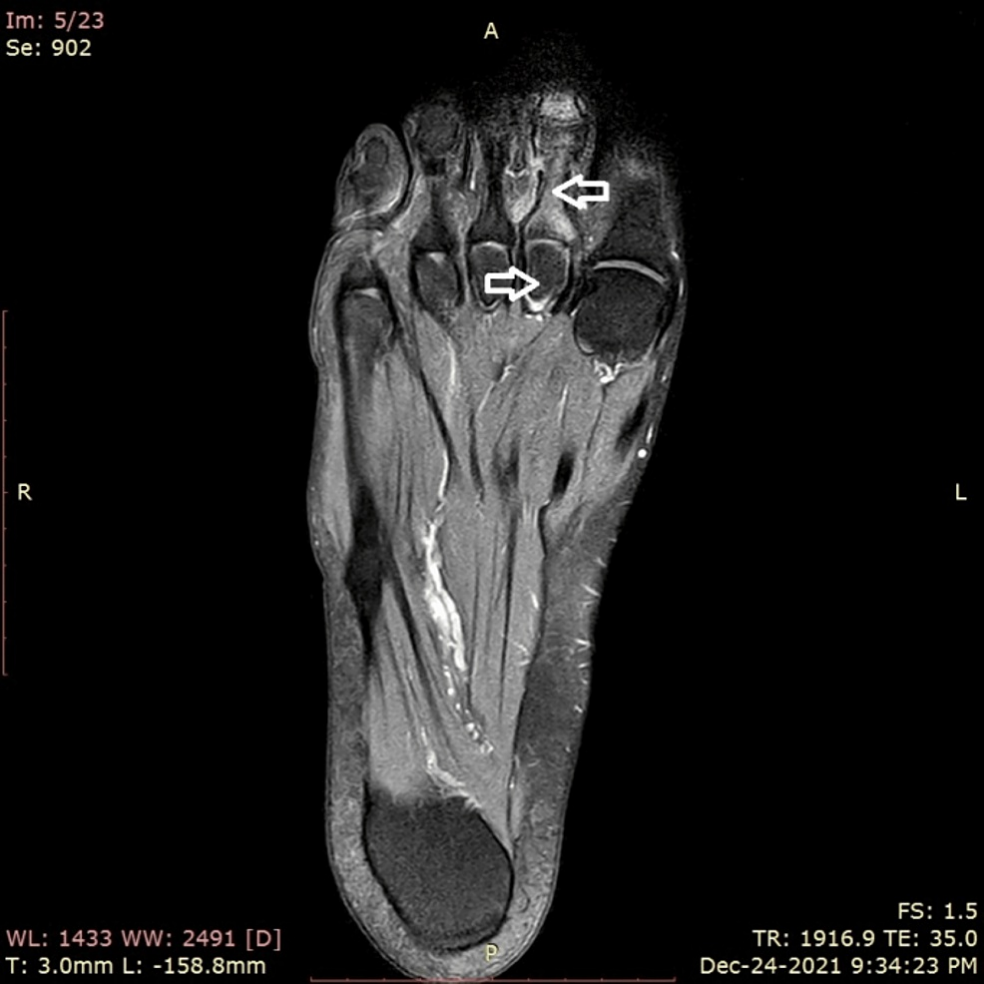
Improvement of the inflammation as compared to the previous MRI (white arrows)

With the findings of sterile osteitis, a diagnosis of SAPHO syndrome was reached. The patient was put on a treatment regimen containing 50 mg of anti-TNF inhibitor (etanercept) via subcutaneous route once a week, 15 mg of DMARD (methotrexate) orally once a week, and prednisolone from 25 to 5 mg/day for six months. A repeat MRI showed improvement (Figure 4), and he was finally maintained on methotrexate, resulting in complete remission with subsequent MRI scans showing significant interval regression of the disease process over the following years.

## Discussion

Disease manifestations

The SAPHO syndrome, a collection of abnormal conditions affecting the skin and bones, consists of a variety of names and descriptions in the literature suggesting its complex nature [8]. It has previously received a great deal of attention in Japan and Western Europe [6]. Skin diseases (PPP, pustular psoriasis, or severe acne), osteomyelitis, and arthritis are all components of this clinical disorder [8]. The two most common presentations of SAPHO syndrome are hyperostosis and osteitis. An excessive bone formation defines as hyperostosis, and inflamed and painful bones are known as osteitis. Either the cortex, the medullary cavity, or both could be involved [6]. As seen in our case, sterile osteitis of the metatarsals was also the initial radiological finding.

Skin manifestations usually include acne and PPP. Acne may include severe forms of acne fulminans, acne conglobata, and hidradenitis suppurativa. PPP manifests as a persistent flare-up of yellowish intradermal sterile pustules on the palms and soles [6].

The chances of acne or PPP accompanying SAPHO syndrome is 55.7% and 18.3%, respectively [4], but the absence of these skin findings does not rule out the possibility of SAPHO syndrome [6]. Similarly, in our case, the patient had mild acne on his forehead with psoriatic plaques unrelated to SAPHO syndrome, but with the presence of sterile osteomyelitis, we were able to reach a definite diagnosis.

The disease in the form of synovitis and arthritis most commonly involves the anterior chest wall especially the sternoclavicular joints with a high percentage of 65-90%. In descending order of involvement, the rest of the joints affected by SAPHO syndrome are the spine, pelvis, long bones, ileum, and mandible [3,9], whereas in our case, the metatarsals and phalanges of the foot were involved, uncharacteristically. Likewise, another case of a 39-year-old male had been reported, previously, to present with an initial finding of osteomyelitis of the phalanges of the hands, which was later diagnosed as SAPHO syndrome [10]. These atypical presentations and a range of symptoms comprising SAPHO syndrome make it a hard case to identify.

Pathogenesis

Its pathogenesis and etiology are still unclear. Genetic, immunological, and infectious causes have been studied to play some role in disease development [4]. Given that the HLA-B27 haplotype is significantly less common in the SAPHO syndrome than in other types of SpA, some studies point to a genetic predisposition involving sites outside of the MHC [4]. A mouse model of chronic multifocal osteomyelitis (CMO) has revealed the existence of a susceptibility gene at chromosome 18 (CMO locus) [11]. There is mounting evidence that the SAPHO syndrome may be caused by an overactive NF-kB response to intestinal bacteria mediated by the NOD2/CARD15 system in the inflammasome which is also linked to Crohn's disease) [11,12].

Inflammatory responses in this syndrome have been linked to IL-1, 8, 17, and 18 and tumor necrosis factor-alpha [2]. TNF may play a role in the onset and propagation of the disease's rheumatic symptoms. The demonstration of TNF overexpression with a focus on mandibular osteitis and the favorable outcomes with TNF antagonists give credibility to this view [12]. Pointing out a probable link between innate and adaptive immunity, high levels of IL-8 and TNF-α produced by polymorph nuclear lymphocytes (PMN) were reported in this syndrome, whereas PMNs help mature B and T cells using IL-8 and IL-18 [11].

A typically innocuous microorganism, *P. acnes*,* *has been reported to activate the body's immune reaction using toll-like receptors 2 and is a commonly isolated commensal in the SAPHO syndrome [12].

The involvement of infections particularly bacterial and viral infections in the etiology of autoimmune diseases has become an established fact over a span of 20 years. Bacterial and viral infections are thought to operate as arthritogenic stimulants that cause a variety of rheumatic diseases. Molecular mimicry suggests that infectious pathogens can both initiate the creation of cross-reacting antibodies and cause the inflammatory "second strike" mediated by Toll-like receptors [13]. There are many examples to support the idea that viruses can provoke auto inflammation like the presence of Epstein-Barr virus in systemic autoimmune diseases [14] or coronaviruses in rheumatoid arthritis [1].

On the other hand, in an Italian group with SAPHO syndrome, a prevalence of antithyroid antibodies could be seen [15]. Some experts have considered incorporating CRMO and SAPHO into the list of autoinflammatory syndromes [16]. Additionally, there are reports according to which COVID-19 exhibits immunological characteristics similar to autoimmune disorders, such as overactivity of mature natural killer cells and CD8+ T cells or increased inflammatory response by exaggerating cytokines like TNF alpha and IL-6 [17]. Incidents of various autoimmune diseases following COVID infection have been mentioned in the literature like the development of GBS, MIS-C, and SLE [1].

On the basis of these expert conclusions, it is safe to assume that the patient in our case could have developed SAPHO syndrome as a post-COVID complication, a first of its kind, due to the abovementioned immune dysregulation caused by viral infections, especially COVID-19.

The patient in our case had a seronegative profile, but his extra-axial joints were involved rather than the typical sacroiliac joints or paravertebral area, although SAPHO is closely related to seronegative spondyloarthropathies on the basis of the involvement of various axial joints like sacroiliac joint or paravertebral involvement.

It also shares various clinical features with CRMO [2]. According to Skrabl-Baumgartner et al., pediatric and adult patients diagnosed with CRMO or SAPHO syndrome had similar clinical features, radiological results, histopathologic findings, and biomarkers. This suggests that these two may be different phenotypes of the same syndrome [18].

Diagnosis

Owing to the overlap of the clinicopathological findings, identification is challenging in cases where there is only one affected bone with a lesion that is frequently mistaken for suppurative osteomyelitis [8]. Schilling and Kessler classified 86 cases of SAPHO syndrome after examining their clinical, radiological, and histological/histopathological data. Depending on the manifestations, they separated these patients into five categories. The disorders in Groups I (SHPP) and III (CRMO) were expected to have clear-cut presentations, whereas Groups II, IV, and V exhibited a spectrum of signs and symptoms (Table 2) [19]. A commonly used diagnostic criterion for SAPHO syndrome, modified in 2003 by Kahn, gives five criteria of which at least one should be met to diagnose a case of SAPHO syndrome. One of these criteria is the presence of an isolated sterile hyperostosis/osteitis of any bone which was present in our case [20].

**Table 2:** Schilling and Kessler’s classification (2000) CRMO: chronic recurrent multifocal osteomyelitis, ACW: anterior chest wall, SCCH: sternocostoclavicular hyperostosis, PPP: palmoplantar pustulosis, SCCH: sternocostoclavicular hyperostosis

Class I	Spondarthritis hyperostotica pustulo-psoriatica	Triad of PPP*+SCCH+productive spondylopathy
Class II	CRMO	Sterile osteomyelitis+PPP*
Class III	ACW syndrome	Anterior chest wall inflammation
Class IV	SCCH	More severe form of Isolated SCCH*
Class V	Acne associated spondyloarthritis	Osteoarticular symptoms in cases of acne pustulosa
*PPP is pustulosis palmoplantaris; SCCH is sternocostoclavicular hyperostosis		

Investigations

Currently, it is not a challenge to identify SAPHO syndrome in cases where there is a typical involvement of bones with accompanying skin manifestation. However, with atypical presentations, where there is no skin involvement or unusual bones are involved, diagnosis is tough and can lead to misdiagnosis [21].

Laboratory findings show normal ESR and CRP that might be raised during disease flare-ups. HLA-B27 is negative in most cases of SAPHO syndrome; it is still relatively more common as compared to the general population with the percentage ranging from 13% to 30% [3]. In our case, these laboratory investigations remained insignificant with a negative HLA-B27 profile.

Imaging like whole-body bone scintigraphy can detect systemic lesions and long bone lesions and is distinctive, but due to its low specificity, other imaging modalities have to be used when required. At very late stages, osteoarticular abnormalities in the long bones can be found by X-ray imaging [20], but its low sensitivity for detecting early changes can delay the diagnosis. Bone hyperplasia can be clearly seen using a CT scan, but it struggles to identify soft tissues [5].

Using MRI, it is possible to distinguish SAPHO syndrome from other conditions including spinal malignancy and infection. It takes superiority over CT scan as it is able to distinguish between active and inactive lesions [21].

The extra-axial locations of bony lesions in this syndrome can lead to a false or missed diagnosis and often depends upon bone biopsy. Bone lesions can have a variety of histologies; however, early lesions can be identified by the presence of polymorphonuclear infiltration, whereas the late phases are marked by lesions with sclerotic bone and obvious marrow fibrosis [6].

In our case, we had also ordered an MRI scan which indicated the presence of osteomyelitis in the MTP and phalanges, and to further prove this point, our patient went under a whole-body bone scintigraphy scan which clearly showed increased pooling of the radiotracer in the same affected area. In addition, bone biopsy and culture followed these investigations to establish sterile osteomyelitis which showed signs of chronic inflammation and no malignancy.

Treatment

The majority of research on SAPHO syndrome treatment to date has been conducted using case reports, case series, or observational cohorts. Randomized clinical trial-based evidence is still insufficient. As a result, there is no agreement on how to treat SAPHO syndrome.

The first line treatment for SAPHO is considered to be NSAIDs, e.g., naproxen (500 mg twice daily), ibuprofen (600 to 800 mg three times daily), or celecoxib 100-200 mg twice daily for a duration of two to four weeks which helps in relieving pain and initial inflammatory symptoms. This is followed by DMARDs that have varying results in different patients like the possible effectiveness of methotrexate 15 to 25 mg orally once weekly in patients with peripheral joint involvement showing improvement in several weeks to months [5]. Based on the available literature, several DMARDs, such as sulfasalazine, hydroxychloroquine, leflunomide, and colchicine, have been suggested to be helpful in SAPHO syndrome as well [4].

Antibiotics namely doxycycline (100 mg twice daily), sulfamethoxazole/trimethoprim (160/800 mg orally twice daily), and clindamycin (400 mg three times a day) have also been reported to play a contributory role in treating SAPHO syndrome, owing to the isolation of *P. acnes* and *Staphylococcus aureus* in many cases and their involvement in pathogenesis [3]. Despite this fact, a trial of vancomycin 1 g intravenously twice a day for six weeks did not bring any clinical or radiological improvement (on subsequent MRI) in our case.

For anti-inflammatory action, bisphosphonates have been previously proven to be effective. As a bridging treatment, corticosteroids can also be used in the treatment for a short span of time in medium doses starting at 10-20 mg daily for two to four weeks [5].

Additionally, many anti-TNF alpha biologics have been demonstrated to be an effective cure for this syndrome, among which infliximab, etanercept, and adalimumab have been listed in previous studies [5]. The preferred anti-TNF alpha biologic in our case was 50 mg etanercept via subcutaneous route once a week which had promising results over the course of six months. Along with this, 15 mg of DMARD (methotrexate) orally once a week, as well as prednisolone from 25 to 5 mg/day, was also given for six months.

With an overall good prognosis, SAPHO syndrome has a relapsing-remitting course with spontaneous resolution. Other cases go through a protracted, occasionally incapacitating, progression marked by the emergence of fresh cutaneous or articular signs [5].

## Conclusions

SAPHO should always be considered an important differential diagnosis in patients with findings like sterile osteomyelitis. As seen in our described case, the skin manifestations were not related to his toe swelling, did not bother him, and were present long before he had COVID, but the presence of a painful inflamed toe and the worsening acne right after COVID highlighted SAPHO syndrome's auto-inflammatory nature allowing it to develop in the background of COVID infections as its potential complication, among many others. SAPHO syndrome’s diagnosis remains challenging as is, especially in the presence of skin lesions that do not lie under the available diagnostic criteria for this syndrome, with a history of Sars-Cov-2 infection. Therefore, it is pertinent that timely multiple imaging modalities should be used for an early and correct diagnosis of this syndrome.
